# Cation Vacancy in Wide Bandgap III‐Nitrides as Single‐Photon Emitter: A First‐Principles Investigation

**DOI:** 10.1002/advs.202100100

**Published:** 2021-07-26

**Authors:** Hang Zang, Xiaojuan Sun, Ke Jiang, Yang Chen, Shanli Zhang, Jianwei Ben, Yuping Jia, Tong Wu, Zhiming Shi, Dabing Li

**Affiliations:** ^1^ State Key Laboratory of Luminescence and Applications, Changchun Institute of Optics, Fine Mechanics and Physics Chinese Academy of Sciences Changchun 130033 China; ^2^ Center of Materials Science and Optoelectronics Engineering University of Chinese Academy of Sciences Beijing 100049 China

**Keywords:** AlGaN, cation vacancy, density functional theory, group theory, single‐photon emitters

## Abstract

Single‐photon sources based on solid‐state material are desirable in quantum technologies. However, suitable platforms for single‐photon emission are currently limited. Herein, a theoretical approach to design a single‐photon emitter based on defects in solid‐state material is proposed. Through group theory analysis and hybrid density functional theory calculation, the charge‐neutral cation vacancy in III‐V compounds is found to satisfy a unique 5‐electron‐8‐orbital electronic configuration with T_*d*_ symmetry, which is possible for single‐photon emission. Furthermore, it is confirmed that this type of single‐photon emitter only exists in wide bandgap III‐nitrides among all the III‐V compounds. The corresponding photon energy in GaN, AlN, and AlGaN lies within the optimal range for transfer in optical fiber, thereby render the charge‐neutral cation vacancy in wide‐bandgap III‐nitrides as a promising single‐photon emitter for quantum information applications.

## Graphical abstract

Neutral cation vacancy in wide bandgap III‐nitrides including AlN, GaN, and AlGaN is predicted to be suitable for single‐photon emission, it has zero phonon line that lies within the optimal range for transmission in optical fiber and a moderate radiative rate, thereby render it as a promising single‐photon emitter for quantum information applications.

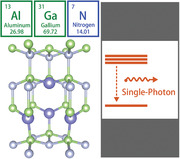

## 1 Introduction

Single‐photon is suitable to serve as a quantum bit (qubit)^[^ ^1^ ^]^ for the encoding, communication, and measurement of quantum information^[^ ^2^ ^]^ since it can travel over long distances while interacting weakly with the environment and can be manipulated with linear optics.^[^ ^3^ ^]^ Single‐photon emitter (SPE) is hence the central building block for many quantum information technologies. The early‐stage SPE was based on single‐atom,^[^ ^4^, ^5^ ^]^ however, it suffered from drawbacks such as low efficiency and reliability. Solid‐state materials including quantum dots^[^ ^6^, ^7^ ^]^ and color centers^[^ ^8^, ^9^ ^]^ with atom‐like isolated levels are promising types of single‐photon sources due to the convenient combination with advanced technologies of the semiconductor industry. The color center, which is a kind of fluorescent defect in solid‐state material with the wavefunction localized on the atomic scale length,^[^ ^10^ ^]^ can potentially realize single‐photon emission at room temperature.^[^ ^11^ ^]^ For instance, the diamond's NV (N_C_V_C_) center,^[^ ^12^ ^]^ SiV (Si_C_V_C_) center;^[^ ^13^ ^]^ the silicon carbide's silicon‐vacancy (V_Si_) center,^[^ ^14^ ^]^ divacancy (V_Si_V_C_) center,^[^ ^15^ ^]^ antisite‐carbon‐vacancy (C_Si_V_C_) center;^[^ ^16^ ^]^ and the zinc oxide's zinc‐vacancy (V_Zn_) center^[^ ^17^ ^]^ have received a lot of investigations.

In recent years, benefits from the mature technique of material growth and device fabrication, III‐V compounds have become commercial semiconductors,^[^ ^18^ ^]^ among them the III‐nitrides that possess wide‐bandgap^[^ ^19^ ^]^ meet the criteria of host material for single‐photon emission. In 2017, Berhane et al. had realized the room‐temperature single‐photon emission in GaN.^[^ ^20^ ^]^ In 2018, Zhou et al. had reported near‐infrared emitters based on GaN with high photon purity.^[^ ^21^ ^]^ In 2020, Xue et al. and Bishop et al. had reported room‐temperature single‐photon emission in AlN,^[^ ^22^, ^23^ ^]^ the antisite‐nitrogen‐vacancy (N_Al_V_N_) and divacancy (V_Al_V_N_) were predicted to be possible sources of the single‐photon signal.^[^ ^22^ ^]^ As the III‐nitrides belong to ionic semiconductor, the dangling bond energy level lies in the lower part of the bandgap for cation vacancy (V_cation_), while it lies in the upper part of the bandgap for anion vacancy (V_anion_).^[^ ^24^ ^]^ It was found that the defect energy level for nitrogen‐vacancy (V_*N*_) in AlN was close to the conduction band maximum (CBM). Transition metal dopant substitution^[^ ^25^ ^]^ and strain‐driven^[^ ^26^ ^]^ strategies were proposed theoretically to adjust the defect electronic configuration to make V_N_ in AlN suitable for single‐photon emission. For V_cation_, since its defect energy level was found close to the valence band maximum (VBM), many theoretical investigations were focused on negatively charged V_cation_, including its light emission^[^ ^27^, ^28^, ^29^ ^]^ and carrier doping^[^ ^30^ ^]^ property. However, whether V_cation_ can realize single‐photon emission by tuning its electronic configuration remains unclear.

**Figure 1:**
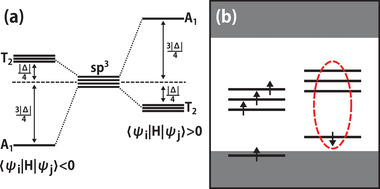
a) The schematic process of sp^3^ dangling bond energy level splitting under *T*_*d*_ symmetry. b) The 5‐electron‐8‐orbital electronic configuration when 〈ψ_*i*_|*H*|ψ_*j*_〉 < 0.

By reviewing the previous reports, we find most of the successful host materials are in wurtzite and zinc‐blende structures, in which all atoms are tetrahedrally coordinated. The four sp^3^ dangling bonds (ψ_*i*_, (*i* = 1, 2, 3, 4)) around a vacancy have the same energy level 〈ψ_*i*_|*H*|ψ_*i*_〉 = *E* and inter‐bond interaction 〈ψ_*i*_|*H*|ψ_*j*_〉 = −Δ/4. Due to the interaction between dangling bonds, the energy level splits into a nondegenerate *A*_1_ state and a triple degenerate *T*_2_ state with an energy difference of Δ under the *T*_*d*_ symmetry. The relative position of *A*_1_ and *T*_2_ depends on the sign of 〈ψ_*i*_|*H*|ψ_*j*_〉 as shown in **Figure** **1**, and it determines the relative energy level for different electronic configurations. For the case of 〈ψ_*i*_|*H*|ψ_*j*_〉 < 0, the many‐electron effect analysis shows that a ‘5‐electron‐8‐orbital’ electronic configuration is suitable for single‐photon emission. Whereas all the defect levels in the spin‐up channel are occupied; in the spin‐down channel, only the low energy *A*_1_ level is occupied, and all the *T*_2_ levels are empty, the optical emission corresponds to the transition of *A*_1_↔*T*_2_ (details are described in the Supporting Information).

In this work, based on group theory analysis and first‐principles computation, we present a comprehensive study of the single‐photon emission property of V_cation_ in III‐V compounds. We find that the charge‐neutral V_cation_ in III‐V compounds can meet the specific 5‐electron‐8‐orbital electronic configuration. Moreover, the charge‐neutral V_cation_ in wide bandgap III‐nitrides including GaN, AlN, AlGaN, and low In component InGaN are thermodynamically stable and can serve as SPE. The corresponding defect energy level, formation energy, and photon energy of the proposed SPE are presented

## 2 Experimental Section

**Figure 2:**
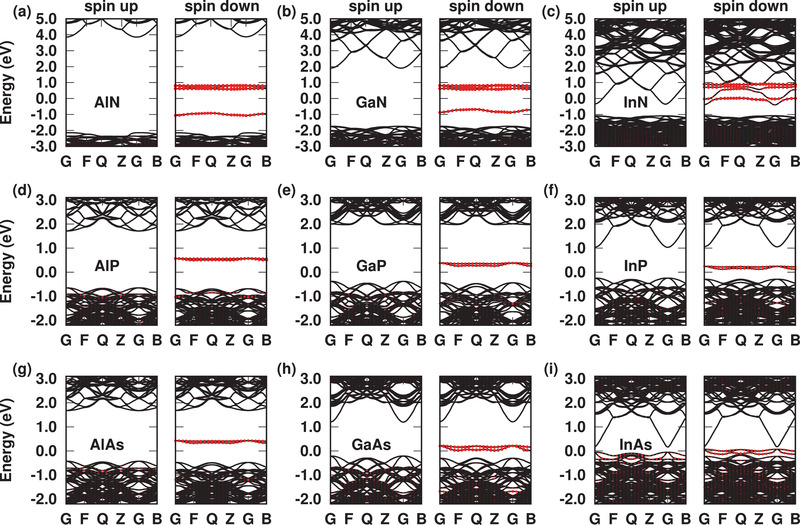
Band structures for neutral V_cation_ in III–V compounds calculated with HSE06 functional, here G (0.0, 0.0, 0.0), F (0.0, 0.5, 0.0), Q (0.0, 0.5, 0.5), Z (0.0, 0.0, 0.5), B (0.5, 0.0, 0.0) refer to the high‐symmetry special points in the first Brillouin zone, the Fermi level is set to zero, the orbital contribution of four anions around V_cation_ is represented by red dots.

It was first characterized whether charge‐neutral V_cation_ in III–V (III = Al, Ga, In; V = N, P, As) compounds satisfy the 5‐electron‐8‐orbital electronic configuration. The band structures calculated with HSE06 hybrid functional are shown in **Figure** **2** (the atomic structures are shown in Figure S3, Supporting Information). Taking the Fermi level as a reference, the VBM position of the host material tends to increase as the group III component varies from Al to In when fixing the group V component or as the group V component varies from N to As when fixing the group III component. The orbital contribution of anions around V_cation_ shows the defect levels of V_cation_ in the spin‐up channel are fully occupied, while in the spin‐down channel the *A*_1_ state lies below the *T*_2_ states and only the *A*_1_ state is occupied. This indicates a negative inter‐bond interaction (〈ψ_*i*_|*H*|ψ_*j*_〉) between the anion dangling bonds, the charge‐neutral V_cation_ in III‐V compounds thus satisfies the 5‐electron‐8‐orbital electronic configuration.

To serve as an SPE, the defect energy level of V_cation_ should lie within the bandgap to make the optical transition do not introduce interference from electronic states of the host material.^[^ ^24^ ^]^ The relative position between the defect energy level of V_cation_ and band edge of host material depends on the energy level of the anion sp^3^ dangling bond, the corresponding symmetry‐induced splitting, and the bandgap of the host material. For V_cation_ in III‐phosphide and III‐arsenide, as shown in Figure 2d–i, the *T*_2_ states in the spin‐down channel are located within the bandgap, while the *A*_1_ state lies below the VBM, this can be attributed to the low sp^3^ level of P/As atom. Because the *T*_2_ states are degenerate and are all unoccupied, the charge‐neutral V_cation_ in III‐phosphide or III‐arsenide is not suitable for single‐photon emission. Since the sp^3^ dangling bond energy of the N atom is the highest one among all the group V elements,^[^ ^31^ ^]^ the possible case that the *A*_1_ level lies above VBM should be III‐nitride. As shown in Figure 2a–c, for V_cation_ in AlN, GaN, and InN, the *A*_1_ level in the spin‐down channel lies above the VBM. However, the bandgap of InN is too narrow, the defect levels of V_cation_ are all above the CBM. While V_cation_ in wide bandgap III‐nitrides of GaN and AlN are suitable for single‐photon emission whereas all the *A*_1_ and *T*_2_ levels lie within the bandgap.

**Figure 3:**
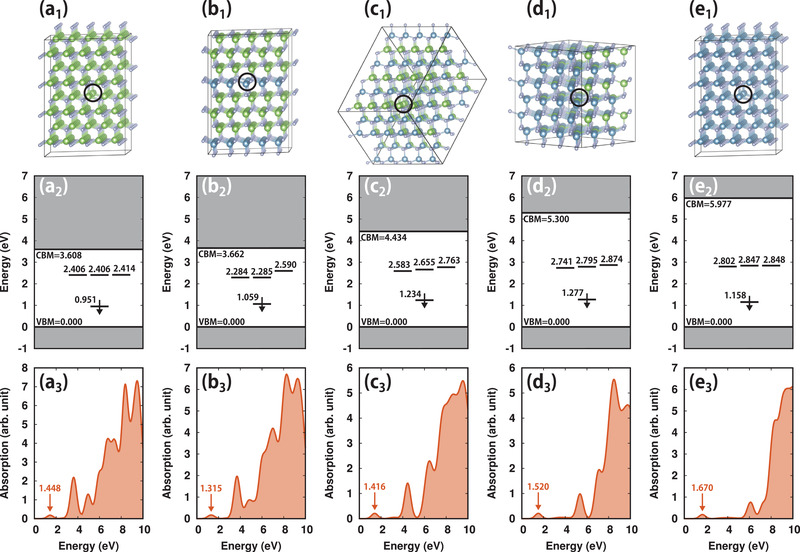
a_1_–e_1_) The atomic supercell structures for V_Ga_ in GaN, and V_Al_ in Al_0.25_Ga_0.75_N, Al_0.5_Ga_0.5_N, Al_0.8_Ga_0.2_N, and AlN, the corresponding defect energy levels in the spin‐down channel (the VBM is set to zero) and absorption spectrum for calculated with HSE06 functional are shown in (a_2_–e_2_) and (a_3_–e_3_), respectively.

Based on our previous analysis, it has been proposed to use charge‐neutral V_cation_ in wide bandgap III‐nitride as a potential SPE. To numerically assess the single‐photon emission property, the defect level of V_cation_ in larger supercells of GaN and AlN was calculated, each includes 399 atoms as shown in **Figure** **3**a_1_,e_1_, with a Γ point only *K*‐mesh sampling. The defect level diagrams of V_cation_ in GaN and AlN are shown in Figure 3a_2_,e_2_. The energy splitting (Δ) between *A*_1_ and *T*_2_ in GaN (1.45 eV) is smaller to that in AlN (1.64 eV). The interatomic distance (see Table S2, Supporting Information) shows that the distance between the N atom around V_cation_ is smaller in AlN, which results in a larger inter‐bond interaction and thereby larger energy splitting (Δ) in AlN. The energy differences between defect level and VBM/CBM in GaN and AlN are all larger than 0.9 eV, indicating the thermal transition from the host material to the defect state is small.

The strain effect on the III‐nitrides epilayer, which can be achieved by mechanical wafer bending experimentally, has been widely investigated. Here the strain effect on the single‐photon emission property of V_cation_ in GaN and AlN was also studied, biaxial strain vertical to the (0001) direction including a 95% compress strain and a 105% tensile strain was considered. The results (see Figure S9, Supporting Information) indicate that for V_cation_ in GaN and AlN, both the band edge position of the host material and the defect energy level change under the extrinsic strain, and they are all located within the bandgap in the considered strain range. For specific, the bandgap of the host material and the energy difference between *A*_1_ and *T*_2_ increase when applying a compress strain, while they decrease when applying a tensile strain. This offers a way to tune the single‐photon emission property of V_cation_ experimentally.

To examine whether the proposed SPE is optically active, the absorption spectrum of V_cation_ was calculated, the results for V_cation_ in GaN and AlN are shown in Figure 3a_3_,e_3_, respectively. Since there are no in‐gap states in the spin‐up channel and the *T*_2_ states are nearly degenerate in the spin‐down channel, the obvious peak below 2 eV corresponds to the transition from *A*_1_ to *T*_2_, this indicates an optical transition can occur between the two states. The small and broad absorption of V_cation_ in AlN in Figure 3e_3_ includes the transition from VBM to *T*_2_ and from *A*_1_ to CBM, the calculated moment matrix |〈ψ_*i*_|*p*|ψ_*j*_〉|^2^ (see Equation S24, Supporting Information) shows that the corresponding magnitude of *A*_1_ ↔ *T*_2_ is about 3 times larger than VBM ↔ *T*_2_ and *A*_1_ ↔ CBM, thus the broad absorption has little effect on the single‐photon emission property. The absorption spectrum for V_cation_ in strained GaN and AlN are shown in Figure S9, Supporting Information, the corresponding absorption peak of V_cation_ blue (red) shifted under a compress (tensile) strain, consistent with the change of the energy difference between *A*_1_ and *T*_2_.

In addition to the binary GaN and AlN, III‐nitride alloy is also widely investigated since it has a tunable bandgap. The single‐photon emission property of V_cation_ in III‐nitride alloy is now calculated, the wurtzite structure of III‐nitride alloy is built by cluster expansion method,^[^ ^32^ ^]^ the corresponding unit cell structures are shown in Figure S1, Supporting Information. The structure of V_cation_ in III‐nitride alloy is determined by directly remove a cation from the perfect alloy, as indicated later, the formation energy of neutral V_cation_ is high, such a non‐equilibrium way is practical to generate V_cation_ in the experimental condition.

For AlGaN alloy as a host material, the atomic structures with low, medium, and high Al composition of 0.25, 0.5, and 0.8 are chosen as representatives. The calculated band structures indicate that the CBM of AlGaN is contributed by the s orbital of the N atom (see Figure S2, Supporting Information). The VBM of AlGaN with low Al composition is contributed by the *p*_*x*_ and *p*_*y*_ orbital of the N atom. For AlGaN with high Al composition, the VBM is mainly contributed by the *p*_*z*_ orbital of the N atom. This results in a different light emission mode in the electrically pumped light emission device,^[^ ^33^ ^]^ this property can be used to separate the light signals from host material and V_cation_ efficiently. The calculated defect energy level and absorption spectrum of V_Al_ in AlGaN with different Al compositions are shown in Figure 3b–d (the results for V_Ga_ in AlGaN are shown in Figure S10, Supporting Information), the qualitative property of defect levels of V_cation_ in AlGaN is the same as that of GaN and AlN. Due to the different interactions between the Al‐N and Ga‐N bond, the N atoms around V_cation_ move unsymmetrically from their original position. Such a symmetry‐lowing effect caused by the random alloy reduces the coupling between sp^3^ dangling bonds and splits the degenerated *T*_2_ states. Also, as shown in Figure 3, the optical transition is allowed between *A*_1_ and *T*_2_ for V_cation_ in AlGaN, the moment matrix (see Equations S19 and S21, Supporting Information) of the broad absorption peak in Figure 3c_3_,d_3_ is similar to the case of V_cation_ in AlN, and it does not affect the single‐photon emission property.

For InGaN alloy, previous calculation showed that V_cation_ was suitable for single‐photon emission in GaN but not InN, which is due to the low CBM position of InN. Since the bandgap of InGaN decreases monotonically with the In component, there should be a maximum In component for V_cation_ to serve as an SPE in InGaN alloy. Therefore, the effect of different In compositions on the single‐photon emission property of V_cation_ is calculated, it is found that for V_cation_ in In_0.25_Ga_0.75_N, the lowest *T*_2_ state of V_cation_ is nearly in resonance with the CBM, in this case, the insulation of the CBM is broken. While for a low In component case of In_0.125_Ga_0.875_N, all the defect energy levels in the spin‐down channel are located within the bandgap (see Figures S4 and S6, Supporting Information). It was concluded that for V_cation_ to realize single‐photon emission in InGaN, a maximum component of In should not exceed about 25%. The calculated defect level and absorption spectrum of V_cation_ in In_0.125_Ga_0.875_N are qualitatively the same as the case of GaN and AlN, the results are shown in Figure S8, Supporting Information.

To quantitatively characterize the single‐photon emission property of V_cation_, the zero‐phonon line (ZPL) was calculated, which is the optical transition energy without the phonon contribution. The excited‐state structure was optimized with a constraint DFT method^[^ ^34^ ^]^ by restricting the excited‐state electronic configuration. The results are listed in **Table** **1**, and the corresponding interatomic distances between anions at ground (excited) state are listed in Table S2, Supporting Information. It can be seen that the ZPL of V_cation_ in pure GaN and AlN shows a monotonic dependence on the strain, it increases (decreases) under the compress (tensile) strain. For AlGaN alloy, though the mixing of Al has a similar monotonic effect on the lattice constant, the local environment for V_cation_ differs a lot, it affects the geometry of ground and excited states and then affects the ZPL. Even in the same AlGaN alloy, the ZPL of V_Al_ and V_Ga_ differs a lot, thus, the ZPL does not show an obvious dependence on the mixing ratio of AlGaN. In recent experimental investigations, the single‐photon emission in GaN with ZPL from 1085 to 1340 nm has been observed,^[^ ^21^ ^]^ based on our ZPL results, the V_cation_ should have contributions to the corresponding single‐photon signal. The ZPL of V_cation_ in these III‐nitrides has a large overlap with the optimal range of ≈1.2−1.6 μm that can reduce the attenuation^[^ ^35^ ^]^ for optical fiber telecommunication. Besides, the calculated radiative lifetime τ_rad_ of V_cation_, as listed in Table 1, are comparable to that of the NV^−^ center in diamond (≈10−30 ns).^[^ ^36^ ^]^ These advantages indicate that V_cation_ is suitable for practical quantum communication application.

**Table 1:** Calculated moment matrix |〈ψ_*i*_|*p*|ψ_*j*_〉|^2^, transition energy Δ*E* between defect levels, radiative lifetime τ_rad_, and ZPL for V_cation_ in GaN, AlN, AlGaN, and InGaN

	|〈ψ_*i*_|*p*|ψ_*j*_〉|^2^ [au]	Δ*E* [eV]	τ_rad_ [ns]	ZPL [eV]
V_Ga_ in 95% strain‐GaN	1.90 × 10^−2^	1.97	14.23	1.49
V_Ga_ in GaN	1.05 × 10^−2^	1.45	35.18	0.70
V_Ga_ in 105% strain‐GaN	6.06 × 10^−3^	1.08	81.56	0.57
V_Al_ in Al_0.25_Ga_0.75_N	9.22 × 10^−3^	1.22	48.59	0.68
V_Ga_ in Al_0.25_Ga_0.75_N	1.31 × 10^−2^	1.30	32.04	0.92
V_Al_ in Al_0.5_Ga_0.5_N	1.61 × 10^−2^	1.35	25.80	0.76
V_*Ga*_ in Al_0.5_Ga_0.5_N	1.31 × 10^−2^	1.25	34.28	0.70
V_*Al*_ in Al_0.8_Ga_0.2_N	1.60 × 10^−2^	1.46	24.72	0.84
V_*Ga*_ in Al_0.8_Ga_0.2_N	1.71 × 10^−2^	1.45	23.29	0.96
V_Al_ in 95% strain‐AlN	2.59 × 10^−2^	2.10	10.83	1.96
V_Al_ in AlN	2.24 × 10^−2^	1.64	16.03	0.94
V_Al_ in 105% strain‐AlN	8.54 × 10^−3^	1.36	50.63	0.72
V_In_ in In_0.125_Ga_0.875_N	7.38 × 10^−3^	1.14	62.78	0.62
V_Ga_ in In_0.125_Ga_0.875_N	4.48 × 10^−3^	1.17	100.63	0.56

**Figure 4:**
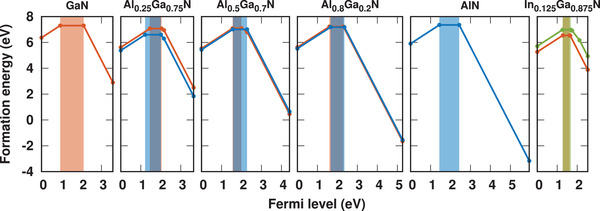
The formation energy of V_cation_ in GaN, AlN, AlGaN, and InGaN at N rich condition, the red, blue, and green lines corresponds to V_Ga_, V_Al_, and V_In_, respectively, the corresponding colored area indicates the Fermi level where neutral V_cation_ is stable.

As one of the key factors for the proposed SPE, the 5‐electron‐8‐orbital electronic configuration should be stable in experimental conditions. Here, the thermodynamic stability of V_cation_ in GaN, AlN, and AlGaN/InGaN alloy was assessed. The formation energies as a function of the Fermi level are shown in **Figure** **4**. It is seen that different charge states of V_cation_ defect can be stable in the bandgap, the neutral V_cation_ is stable in the lower part in the bandgap, indicating the p‐type III‐nitrides is requested to guarantee the 5‐electron‐8‐orbital configuration. Recently, through using superlattice doping,^[^ ^37^ ^]^ polarization‐induced doping^[^ ^38^ ^]^ techniques, etc., the p‐type doping efficiency in III‐nitrides can be effectively enhanced, it is possible to take advantage of these carrier doping techniques to make charge‐neutral V_cation_ thermodynamically stable. However, as an impurity, the dopant may affect the property of SPE, the effect of Mg_cation_ as an example was tested, the results show the major property of SPE will not be affected by the cation dopant as long as the structure and local electronic configuration of V_cation_ is maintained (see Figures S5 and S7, Supporting Information). For practical application, since the atomic structure of V_cation_ is simple, there are various techniques to achieve it such as electron irradiation,^[^ ^39^ ^]^ and pulse laser irradiation,^[^ ^40^ ^]^ the SPE based on V_cation_ is thus achievable in experimental conditions.

For the SPE in bulk material, refraction is an issue that strongly influences the signal extracting. In practice, recent structures of III‐nitrides generally have a size of less than a few hundred nanometers, which is far less than the photon wavelength, the refraction issue is thus irrelevant. Since the mono vacancy structure possesses no inversion symmetry, the external field induces charge fluctuation and causes spectral diffusion,^[^ ^41^ ^]^ however, its magnitude is not necessarily large for a single vacancy as indicated in a recent theoretical study.^[^ ^42^ ^]^ The reported SPE here is only applicable in GaN and AlN among all the III‐V compounds, where the inter‐bond interaction 〈ψ_*i*_|*H*|ψ_*j*_〉 is negative, and the *A*_1_ state lies below *T*_2_. As indicated in Figure 1, when 〈ψ_*i*_|*H*|ψ_*j*_〉 > 0 the *T*_2_ states lies below *A*_1_, which results in a different electronic configuration, further numerical calculations of vacancy with a T_*d*_ symmetry in other material is a promising way to searching new SPE.

## 3 Conclusion

In summary, we have symmetrically investigated the single‐photon emission property of V_cation_ in the III‐V compound. Based on the group theory analysis and first‐principles calculation with hybrid density functional, we predict that the charge‐neutral V_cation_ in III‐V compounds has a unique 5‐electron‐8‐orbital electronic configuration, which is suitable for single‐photon emission. Furthermore, we confirm that the charge‐neutral V_cation_ can only serve as SPE in wide bandgap III‐nitrides among all the III‐V (III = Al, Ga, In; V = N, P, As) compounds. The charge‐neutral V_cation_ in AlN, GaN, and III‐nitride alloy is thermodynamically stable and the corresponding ZPL lies within the optimal range for low‐loss fiber transmission, which makes this type of SPE particularly useful in practical applications. Our investigation also sheds light on the concept of designing an SPE with a precisely tuned electronic configuration.

## Conflict of Interest

The authors declare no conflict of interest.

## Supporting information

Supporting Information

## Data Availability

Research data are not shared.
