# Supplementary material for: Cation Vacancy in Wide Bandgap III‐Nitrides as Single‐Photon Emitter: A First‐Principles Investigation
Source: Adv Sci (Weinh). 2021 Jul 26;8(18):2100100. doi: 10.1002/advs.202100100 (PMC8456231; doi:10.1002/advs.202100100)
Supplement: Supplementary file 1 — Supporting Information [file ADVS-8-2100100-s001.pdf]

© 2021 Wiley-VCH GmbH

ADVANCED SCIENCE
Open Access

Supporting Information

for *Adv. Sci.*, DOI: 10.1002/advs.202100100

Cation Vacancy in Wide Bandgap III-Nitrides as Single-Photon Emitter: A First-Principles Investigation

Hang Zang, Xiaojuan Sun, Ke Jiang, Yang Chen, Shanli Zhang, Jianwei Ben, Yuping Jia, Tong Wu, Zhiming Shi, Dabing Li**

Supporting Information
Cation Vacancy in Wide Bandgap III-Nitrides as Single-Photon Emitter: A First-Principles Investigation

Hang Zang Xiaojuan Sun Ke Jiang Yang Chen Shanli Zhang Jianwei Ben Yuping Jia Tong Wu Zhiming Shi Dabing Li**

Dr. H. Zang, Prof. X.-J. Sun, Dr. K. Jiang, Dr. Y. Chen, S.-L. Zhang, Dr. J.-W. Ben, Dr. Y.-P. Jia, T. Wu, Prof. Z.-M. Shi, Prof. D.-B. Li
State Key Laboratory of Luminescence and Applications, Changchun Institute of Optics, Fine Mechanics and Physics, Chinese Academy of Sciences, Changchun 130033, China
E-mail: shizm@ciomp.ac.cn; lidb@ciomp.ac.cn
Prof. D.-B. Li
Center of Materials Science and Optoelectronics Engineering, University of Chinese Academy of Sciences, Beijing 100049, China

1 Group theory and many-electron effect analysis of the vacancy defect

We first use an analytic model to understand the defect energy level and the corresponding many-electron configuration. For a vacancy with T_d symmetry, the Hamiltonian H that projected on sp^3 dangling bond orbitals $\psi_i (i = 1, 2, 3, 4)$ can be written as

$$\begin{pmatrix} E & -\frac{\Delta}{4} & -\frac{\Delta}{4} & -\frac{\Delta}{4} \\ -\frac{\Delta}{4} & E & -\frac{\Delta}{4} & -\frac{\Delta}{4} \\ -\frac{\Delta}{4} & -\frac{\Delta}{4} & E & -\frac{\Delta}{4} \\ -\frac{\Delta}{4} & -\frac{\Delta}{4} & -\frac{\Delta}{4} & E \end{pmatrix} \tag{S1}$$

The dangling bond orbital basis is not orthogonal and there is an overlap integral $S = \langle\psi_i|\psi_j\rangle (i \neq j)$. The calculated eigenvalues are

$$\begin{aligned} E_{A_1} &= \langle v|H|v\rangle = \frac{1}{1+3S}(E - \frac{3\Delta}{4}) \\ E_{T_2} &= \langle t_\alpha|H|t_\alpha\rangle = \frac{1}{1-S}(E + \frac{\Delta}{4}), (\alpha = x, y, z) \end{aligned} \tag{S2}$$

where A_1 is a nondegenerate state, the wavefunction can be expressed as

$$v = \frac{1}{2\sqrt{1+3S}}(\psi_1 + \psi_2 + \psi_3 + \psi_4) \tag{S3}$$

and T_2 is a triple degenerate state, the corresponding wavefunctions are

$$\begin{aligned} t_x &= \frac{1}{2\sqrt{1-S}}(\psi_1 + \psi_2 - \psi_3 - \psi_4) \\ t_y &= \frac{1}{2\sqrt{1-S}}(-\psi_1 + \psi_2 + \psi_3 - \psi_4) \\ t_z &= \frac{1}{2\sqrt{1-S}}(\psi_1 - \psi_2 + \psi_3 - \psi_4) \end{aligned} \tag{S4}$$

For the case that $S \ll 1$, the energy splitting between A_1 and T_2 is given as Δ, the relative position of A_1 and T_2 depends on the sign of Δ, i.e. the sign of $\langle\psi_i|H|\psi_j\rangle$.

To assess the many-electron effect on the energy level of the defect center with different electronic configuration, we build the Slater determinant wavefunction $|\ldots mn\ldots\rangle$, where the basis $|m\rangle$, $|n\rangle$ are composed by $A_1(v)$ and $T_2(t_x, t_y, t_z)$. The total energy is expressed as $\langle\ldots mn\ldots|H|\ldots mn\ldots\rangle$. The electron Hamiltonian can be separated into two parts $H = O_1 + O_2$, where $O_1 = \sum_i h_i = \sum_i(-\frac{1}{2}\nabla_i + \sum_Z \frac{Z}{R_{iZ}})$ represents the kinetic energy and the electrostatic potential induced by ions, $O_2 = \sum_i \sum_{(j<i)} \frac{1}{r_{ij}}$ represents the electron-electron interaction. The total energy can be expressed as

$$
\begin{aligned}
\langle\ldots mn\ldots|H|\ldots mn\ldots\rangle &= \langle\ldots mn\ldots|O_1 + O_2|\ldots mn\ldots\rangle \\
&= \sum_m \langle m|h|m\rangle + \frac{1}{2}\sum_{mn}\Big(\langle mn|mn\rangle - \langle mn|nm\rangle\Big)
\end{aligned}
\tag{S5}
$$

Under the tight-bonding approximation that neglecting all the overlap integrals, the one-electron integrations can be expressed as

$$
\begin{aligned}
\langle v|h|v\rangle &= E - \frac{3}{4}\Delta \\
\langle t_\alpha|h|t_\alpha\rangle &= E + \frac{1}{4}\Delta, (\alpha = x, y, z)
\end{aligned}
\tag{S6}
$$

The two-electron integrals can be expressed as

$$
\left.\begin{matrix}
\langle vv|vv\rangle \\
\langle t_\alpha t_\alpha|t_\alpha t_\alpha\rangle \\
\langle vv|t_\alpha t_\alpha\rangle \\
\langle t_\alpha t_\alpha|t_\beta t_\beta\rangle
\end{matrix}\right\} = \frac{J + 3J'}{4}
\tag{S7}
$$

$$
\left.\begin{matrix}
\langle vt_\alpha|vt_\alpha\rangle \\
\langle t_\alpha t_\beta|t_\alpha t_\beta\rangle \\
\langle vt_y|t_x t_z\rangle
\end{matrix}\right\} = \frac{J - J'}{4} = \frac{U}{4}
\tag{S8}
$$

where J and J' are the two-electron integrals of the dangling bond orbital basis ψ_i

$$
\begin{aligned}
\langle\psi_i\psi_i|\psi_i\psi_i\rangle &= J \\
\langle\psi_i\psi_i|\psi_j\psi_j\rangle &= J'
\end{aligned}
\tag{S9}
$$

Table S1: The symmetry and energy level for different electronic configuration.

Configuration	State	Degeneracy	Wave function	Relative energy
v^2t^3	$^4A_1$	1	$\|v\bar{v}xyz\rangle$	$-\frac{3}{4}\Delta - \frac{3}{2}U$
	^2E	2	$\frac{1}{\sqrt{2}}(\|v\bar{v}x\bar{y}z\rangle - \|v\bar{v}xy\bar{z}\rangle)$	$-\frac{3}{4}\Delta - \frac{3}{4}U$
	$^2T_1$	3	$\frac{1}{\sqrt{2}}(\|v\bar{v}xy\bar{y}\rangle - \|v\bar{v}xz\bar{z}\rangle)$	$-\frac{3}{4}\Delta - \frac{5}{4}U$
	$^2T_2$	3	$\frac{1}{\sqrt{2}}(\|v\bar{v}xy\bar{y}\rangle + \|v\bar{v}xz\bar{z}\rangle)$	$-\frac{3}{4}\Delta - \frac{3}{4}U$
vt^4	$^4T_1$	3	$\|vx\bar{x}yz\rangle$	$\frac{1}{4}\Delta - \frac{3}{4}U$
	$^2A_1$	1	$\frac{1}{\sqrt{3}}(\|vx\bar{x}y\bar{y}\rangle + \|vy\bar{y}z\bar{z}\rangle + \|vz\bar{z}x\bar{x}\rangle)$	$\frac{1}{4}\Delta - \frac{1}{2}U$
	^2E	2	$\frac{1}{\sqrt{6}}(\|vx\bar{x}y\bar{y}\rangle + \|vx\bar{x}z\bar{z}\rangle - 2\|vy\bar{y}z\bar{z}\rangle)$	$\frac{1}{4}\Delta - \frac{5}{4}U$
	$^2T_1$	3	$\frac{1}{\sqrt{6}}(2\|\bar{v}x\bar{x}yz\rangle - \|vx\bar{x}\bar{y}z\rangle - \|vx\bar{x}y\bar{z}\rangle)$	$\frac{1}{4}\Delta - \frac{3}{4}U$
	$^2T_2$	3	$\frac{1}{\sqrt{2}}(\|vx\bar{x}\bar{y}z\rangle - \|vx\bar{x}y\bar{z})$	$\frac{1}{4}\Delta - \frac{3}{4}U$
t^5	$^2T_2$	3	$\|xy\bar{y}z\bar{z}\rangle$	$\frac{5}{4}\Delta - U$

The symmetry of the wavefunction of v^pt^{n-p} belongs to $(A_1 \otimes\ A_1\ldots) \otimes (T_2 \otimes T_2\ldots)$. Based on the direct product table of the representations of the T_d symmetry group, the corresponding configuration with the same symmetry can be obtained. The configuration should also be an eigenstate of the total spin S. For

the charge-neutral cation vacancy of III-V compounds, there are 5 electrons in the defect center, the energies for different 5-electron-8-orbital configurations are shown in Table S1. For the case of $\Delta > 0$ and $U > 0$, the configuration of $|v\bar{v}xyz\rangle$ with three single electrons has the lowest energy.

2 Computational details

2.1 Cluster expansion calculation of alloy structure

The cluster expansion (CE) method is adopted to generate the AlGaN/InGaN alloy with different Al/In components. The AlGaN/InGaN supercells are constructed by the alloy-theoretic-automated toolkit.[1] The cross-validation score is used as a measurement to evaluate the quality of the cluster expansion.[2]

$$\frac{1}{M}\sum_{i}^{M}(E_i^{DFT} - E_i^{CE})^2 \tag{S10}$$

E_i^{CE} is the energy predicted by CE based on $M-1$ structures, E_i^{DFT} is the corresponding energy from the first-principles calculation. Here, for AlGaN alloy, $M = 122$, the supercells include at most 20 atoms, the cross-validation score is 6.8 meV/atom; for InGaN alloy, $M = 108$, the supercells include at most 28 atoms, the cross-validation score is 11.3 meV/atom; for InGaN alloy with at most 25% In component, $M = 62$, the supercells include at most 96 atoms, the cross-validation score is 17.6 meV/atom. The results indicate that the cluster expansions are well-converged. For the DFT calculation, the generalized gradient approximation (GGA) exchange-correlation functional of Perdew-Burke-Ernzerhof (PBE)[3] in combination with the projected augmented wave (PAW) basis was employed for geometrical optimization by using the Vienna *ab initio* simulation package (VASP),[4, 5] an energy cutoff of 500 eV is employed, and the atomic positions are optimized using the conjugate gradient scheme until the maximum force on each atom is less than 0.01 eV/Å. The selected structures of AlGaN/InGaN alloy with different Al/In compositions are shown in Figure S1.

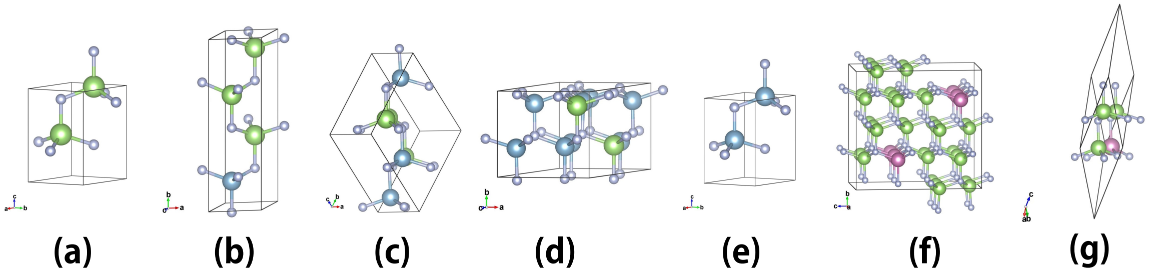

Figure S1: (a)-(g) correspond to the unit cell of GaN, $Al_{0.25}Ga_{0.75}N$, $Al_{0.5}Ga_{0.5}N$, $Al_{0.8}Ga_{0.2}N$, AlN, $In_{0.125}Ga_{0.875}N$, and $In_{0.25}Ga_{0.75}N$, respectively.

2.2 Electronic structure calculation

The atomic positions were optimized until the maximum force on each atom was less than 0.01 eV/Å with PBE functional, and the electronic structures are calculated with the Heyd-Scuseria-Ernzerhof (HSE06)[6] hybrid functional by using the PWmat[7, 8] package. The mixing parameter of the explicit Hartree-Fock exchange energy is set to be 0.32 for AlN, GaN, and GaAs, 0.25 for InN, AlP, GaP, InP, AlAs, and InAs. According to the previous theoretical study, the hybrid functional is accurate for the transitions between internal defect levels.[9] The ONCV-PWM pseudo potential[10] with an energy cutoff of 50 Ry is adopted. For all the calculations, the Al ($3s^2 3p^1$), Ga ($4s^2 4p^1$), In ($5s^2 5p^1$), N ($2s^2 2p^3$), P($3s^2 3p^3$), As ($4s^2 4p^3$), and Mg ($2s^2 2p^6 3s^2$) are treated as valence electrons, and the spin polarization effect is considered. The calculated bandgap for wurtzite InN, GaN, and AlN are 0.69 eV, 3.59 eV, and 5.95 eV; the calculated bandgap for zinc blende InP, GaP, and AlP are 1.27 eV, 2.39 eV, and 2.37 eV; the calculated bandgap for zinc blende InAs, GaAs, and AlAs are 0.21 eV, 1.40 eV, and 2.11 eV, which are consistent with experimental results.[11, 12]

The calculated band structures and the corresponding orbital contributions for AlGaN with different Al ratios are shown in Figure S2. For wurtzite GaN and AlN, the conduction band minimum is contributed by the *s* state of the N atom. The valence band maximum states are more complex. In the absence of

spin-orbit interaction, the top of the valance states at the Γ point is split into a nondegenerate crystal-field split hole (CH) state (contributed by N_{pz} orbital) and a twofold-degenerate state (contributed by N_{px} and N_{py} orbitals) due to the non-cubic crystal-field splitting Δ_{cr}. With the presence of spin-orbit interaction Δ_{so}, the twofold-degenerate state is further split into the heavy hole (HH) and light hole (LH) states. Theoretical and experimental investigations indicate that the order of these states is different between GaN and AlN[13, 14, 12]. Here, the calculated crystal-field splitting energy Δ_{cr} for GaN and AlN are 29 meV and -222 meV, respectively. Previous theoretical calculations of Δ_{cr} for GaN and AlN are 42 meV and -217 meV [14], this indicates that the HSE06 functional can correctly describe the band structure of GaN and AlN.

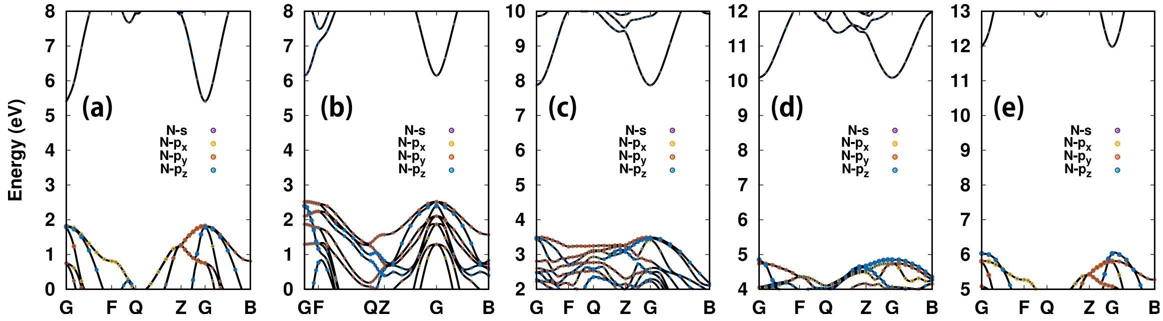

Figure S2: (a)-(e) correspond to the computed fatband structure of GaN, $Al_{0.25}Ga_{0.75}N$, $Al_{0.5}Ga_{0.5}N$, $Al_{0.8}Ga_{0.2}N$, and AlN, respectively; the s, p_x, p_y, and p_z of N atom are marked as purple, yellow, red, and blue dots; here G (0.0, 0.0, 0.0), F (0.0, 0.5, 0.0), Q (0.0, 0.5, 0.5), Z (0.0, 0.0, 0.5), B (0.5, 0.0, 0.0) refer to the high-symmetry spectial points in the first Brillouin zone.

2.3 Absorption spectra and radiative lifetime

The absorption spectrum was calculated with the random phase approximation (RPA) method, which is based on direct Fermi's Golden rule,

$$\epsilon_2(\omega) = \sum_{i \in VB, j \in CB} |\langle \psi_i | \frac{\partial H}{\partial k} | \psi_j \rangle|^2 \delta(E_j - E_i - \omega) \tag{S11}$$

here, $\partial H/\partial k$ is the momentum operator, E_i is the eigenvalue of state $|\psi_i\rangle$.

The radiative lifetime is calculated by Fermi's Golden rule,[15] the concrete form of the radiative rate is given by

$$W_{rad}(\omega) = \frac{\omega^3 n |\mu_{ij}|^2}{3\pi\epsilon_0 \hbar c^3} \tag{S12}$$

where ω is the frequency of emission photon, n is the index of refraction, we use the value n of 2.16, 2.38, and 2.59 for AlN, GaN, and InN, a linear combination of n is used for the alloy, μ_{ij} is the transition dipole moment, ϵ_0 is the vacuum permittivity, $\hbar$ is the reduced Planck constant, c is the vacuum speed of light. According to the relationship of $|\mu_{ij}|^2 = |\langle\psi_i|r|\psi_j\rangle|^2 = |\langle\psi_i|p|\psi_j\rangle|^2 \times \frac{\hbar^2}{\Delta E_{ij}^2 m_e}$, the radiative rate $W_{rad}(\omega)$ and radiative lifetime $\tau_{rad} = 1/W_{rad}(\omega)$ can be obtained.

2.4 Thermodynamic stability calculation for point defect

The formation energies of defects are defined as

$$\Delta H(\alpha, q) = E(\alpha, q) - E(host) + \sum_i n_i \mu_i + q[E_{VBM}(host) + E_F + \Delta V] \tag{S13}$$

here α is the defect type, q is its charge, $E(\alpha, q)$ is the supercell energy with the defect α with charge q, $E(host)$ is the supercell energy without defect, n_i is the number of element i changed from defect α, μ_i

is the corresponding chemical potential, $E_{VBM}(host)$ is the host valence band maximum (VBM) eigenenergy, E_F is the Fermi energy relative to $E_{VBM}(host)$. $\Delta V = V(\alpha, q, R) - V(host, R)$, where R is a place far away from the defect.[16] This formation energy can be used to estimate the defect concentration under the equilibrium condition. Based on Eq. S13, the formation energy of neutral defects does not change with E_F, while for the charged defects, the formation energy varies linearly with E_F and the slope is equal to the charge state q. The calculated chemical potentials for binary and elemental phases are $\mu(AlN)$ = -327.59 eV, $\mu(GaN)$ = -345.87 eV, $\mu(InN)$ = -333.00 eV, $\mu(Al)$ = -56.33 eV, $\mu(Ga)$ = -76.28 eV, $\mu(In)$ = -65.02 eV, $\mu(N)$ = -268.73 eV. Under the N rich condition, $\mu(Al)$ = -58.86 eV, $\mu(Ga)$ = -77.14 eV, $\mu(In)$ = -64.28 eV, $\mu(N)$ = -268.73 eV.

2.5 Atomic defect structure for band structure calculation

The atomic structures of V_{cation} in III-V compounds are shown in Figure S3. For V_{cation} in III-phosphide and III-arsenide, a zinc-blende type supercell containing 215 atoms is used with a (1×1×1) K-point sampling; for V_{cation} in III-nitride, a wurtzite type supercell containing 191 atoms is used with a (2×2×1) K-point sampling.

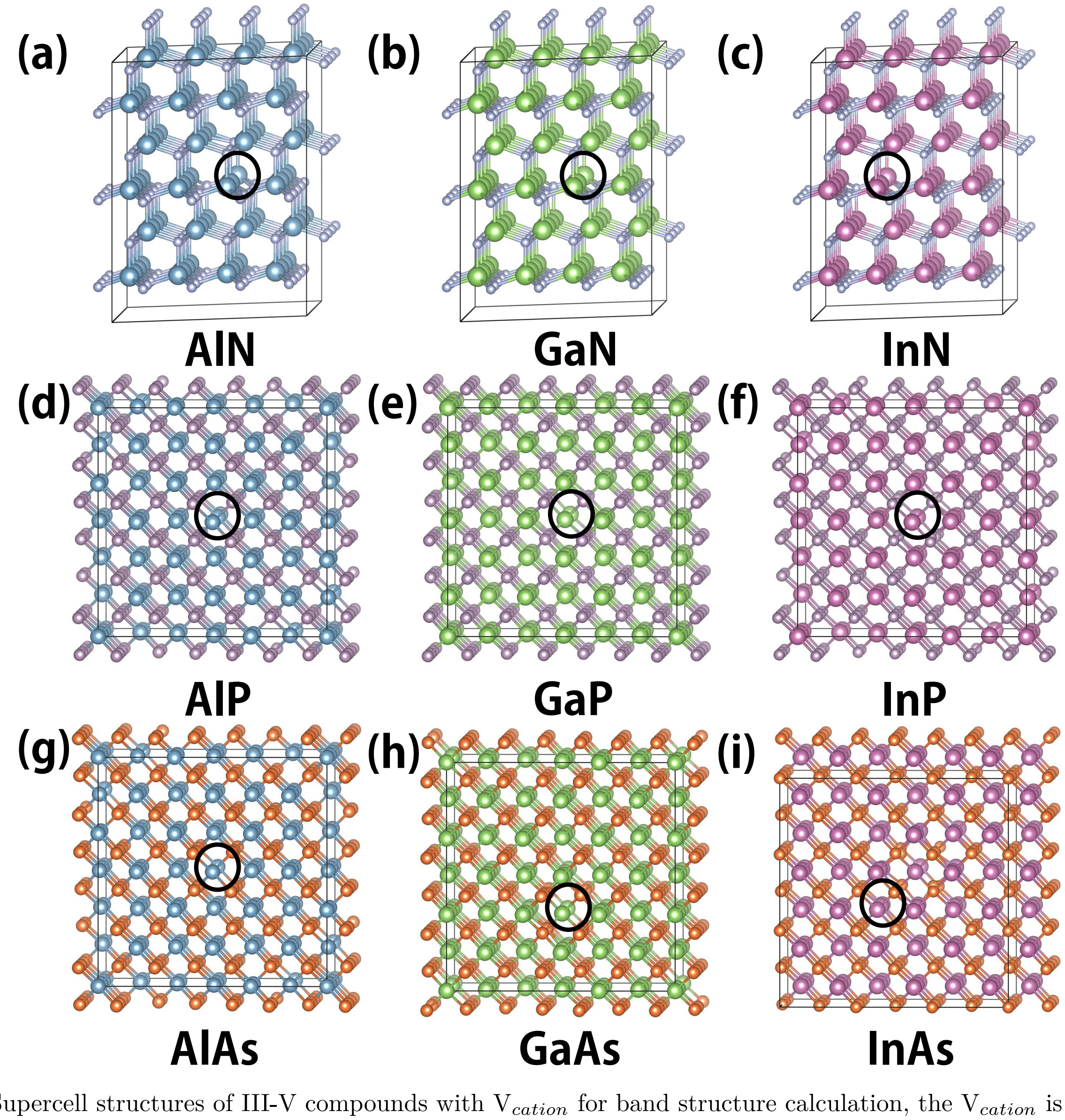

Figure S3: Supercell structures of III-V compounds with V_{cation} for band structure calculation, the V_{cation} is highlighted by the black circle. The blue, green, pink, gray, purple, and orange balls represent Al, Ga, In, N, P, and As atoms, respectively.

The atomic structures of V_{cation} in InGaN alloy are shown in Figure S4, a (1×2×1) mesh is chosen for K-point sampling for $In_{0.125}Ga_{0.875}N$ 127-atom supercell, a (1×2×2) mesh is chosen for K-point sampling for $In_{0.25}Ga_{0.75}N$ 215-atom supercell.

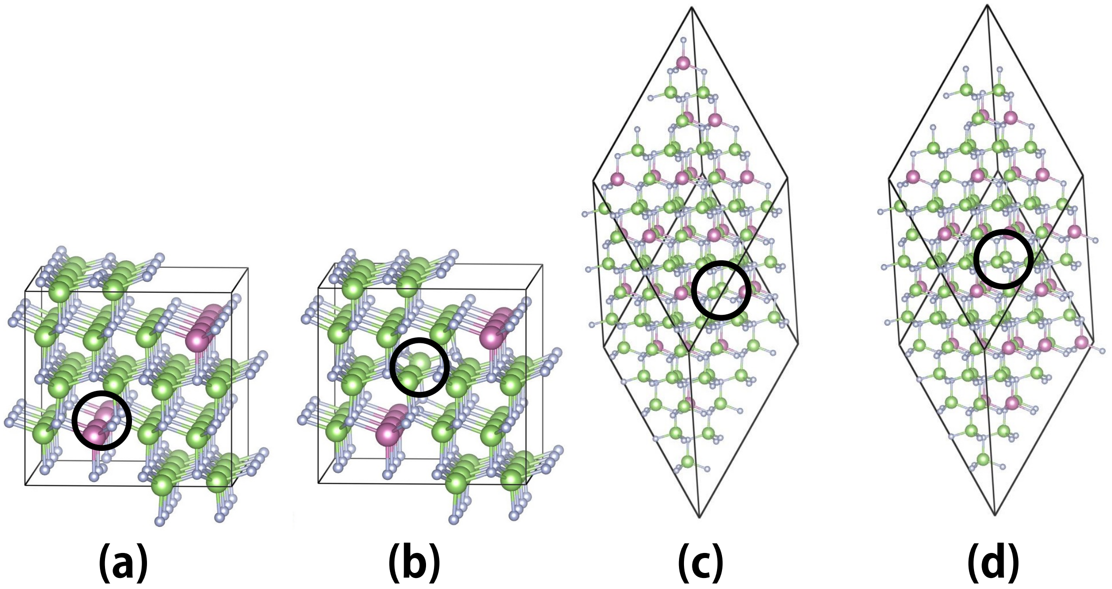

Figure S4: Supercell structure of (a) V_{In} in $In_{0.125}Ga_{0.875}N$, (b) V_{Ga} in $In_{0.125}Ga_{0.875}N$, (c) V_{In} in $In_{0.25}Ga_{0.75}N$, (d) V_{Ga} in $In_{0.25}Ga_{0.75}N$ for band structure calculation, the V_{cation} is highlighted by the black circle. In, Ga, N atoms are represented by pink, green, and gray balls, respectively.

The atomic structures of V_{cation}+Mg_{cation}/Mg^{-}_{cation} in GaN/AlN are shown in Figure S5, a (2×2×1) mesh is chosen for K-point sampling for GaN/AlN 191-atom supercell.

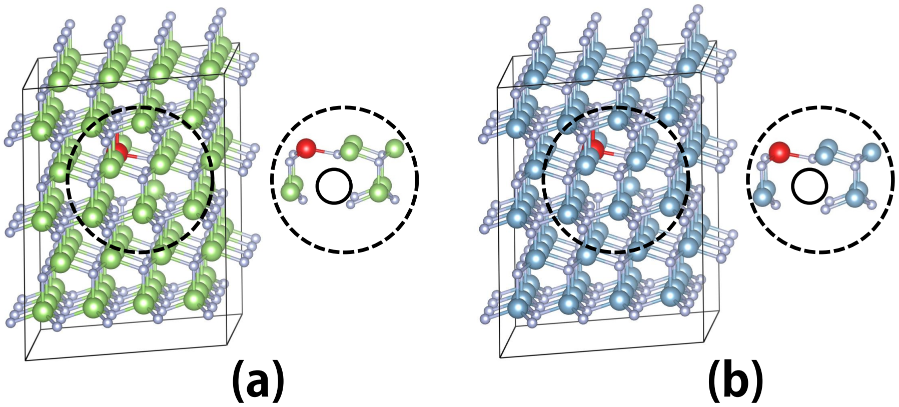

Figure S5: Supercell structure of (a) V_{Ga}+Mg_{Ga}/Mg^{-}_{Ga} in GaN and (b) V_{Al}+Mg_{Al}/Mg^{-}_{Al} in AlN for band structure calculation, the detail of the local structures are shown by the dashed circle, the V_{cation} is highlighted by the black circle. Ga, Al, Mg, N atoms are represented by green, blue, red, gray balls, respectively.

3 Additional computation results

3.1 Band structure of V_{cation}

The band structure of V_{cation} in InGaN calculated with HSE06 functional is shown in Figure S6, the supercell structures are shown in Figure S4.

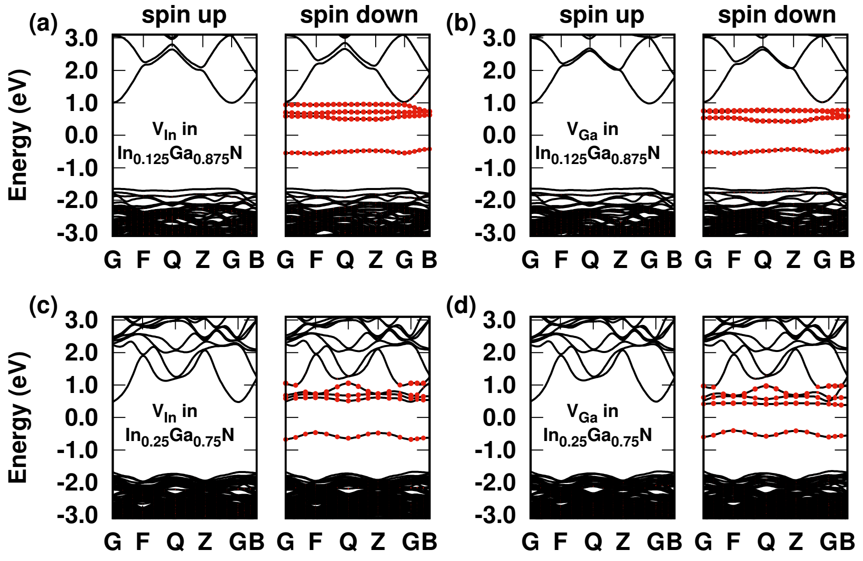

Figure S6: Band structures for neutral V_{cation} in InGaN calculated with HSE06 functional, here G (0.0, 0.0, 0.0), F (0.0, 0.5, 0.0), Q (0.0, 0.5, 0.5), Z (0.0, 0.0, 0.5), B (0.5, 0.0, 0.0) refer to the high-symmetry special points in the first Brillouin zone, the Fermi level is set to zero, the orbital contribution of four anions around V_{cation} is represented by red dots.

The band structure of V_{cation}+Mg_{cation}/Mg^{-}_{cation} in GaN/AlN calculated with HSE06 functional is shown in Figure S7, the supercell structures are shown in Figure S5.

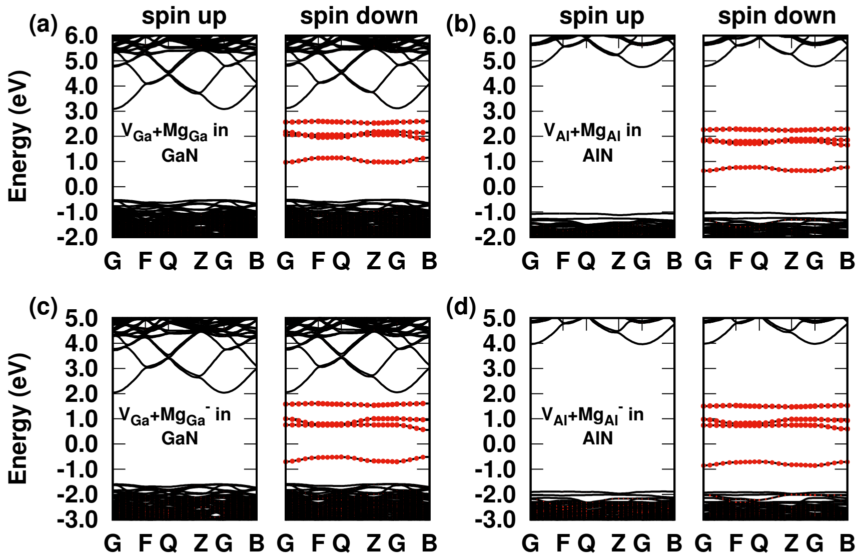

Figure S7: Band structures for V_{cation}+Mg_{cation}/Mg^{-}_{cation} in GaN and AlN calculated with HSE06 functional, here G (0.0, 0.0, 0.0), F (0.0, 0.5, 0.0), Q (0.0, 0.5, 0.5), Z (0.0, 0.0, 0.5), B (0.5, 0.0, 0.0) refer to the high-symmetry special points in the first Brillouin zone, the Fermi level is set to zero, the orbital contribution of four anions around V_{cation} is represented by red dots.

3.2 Defect energy level and absorption spectrum for V_{cation}

The defect energy level and absorption spectrum of V_{cation} in $In_{0.125}Ga_{0.875}N$ calculated with HSE06 functional are shown in Figure S8, a 255-atom supercell is used with a Γ point only K mesh sampling.

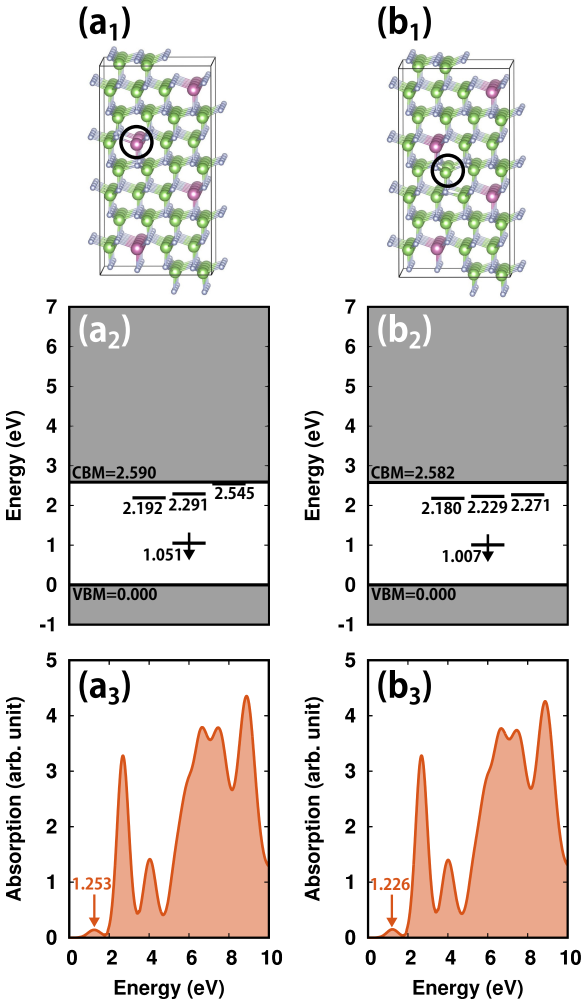

Figure S8: (a_1)-(b_1) are the atomic supercell structures for V_{In} in $In_{0.125}Ga_{0.875}N$ and V_{Ga} in $In_{0.125}Ga_{0.875}N$, the corresponding defect energy levels in the spin-down channel (the VBM is set to zero) and absorption spectrum calculated with HSE06 functional are shown in (a_2)-(b_2) and (a_3)-(b_3), respectively.

The defect energy level and absorption spectrum of V_{cation} in strained GaN/AlN calculated with HSE06 functional are shown in Figure S9, a 399-atom supercell is used with a Γ point only K mesh sampling for both GaN and AlN.

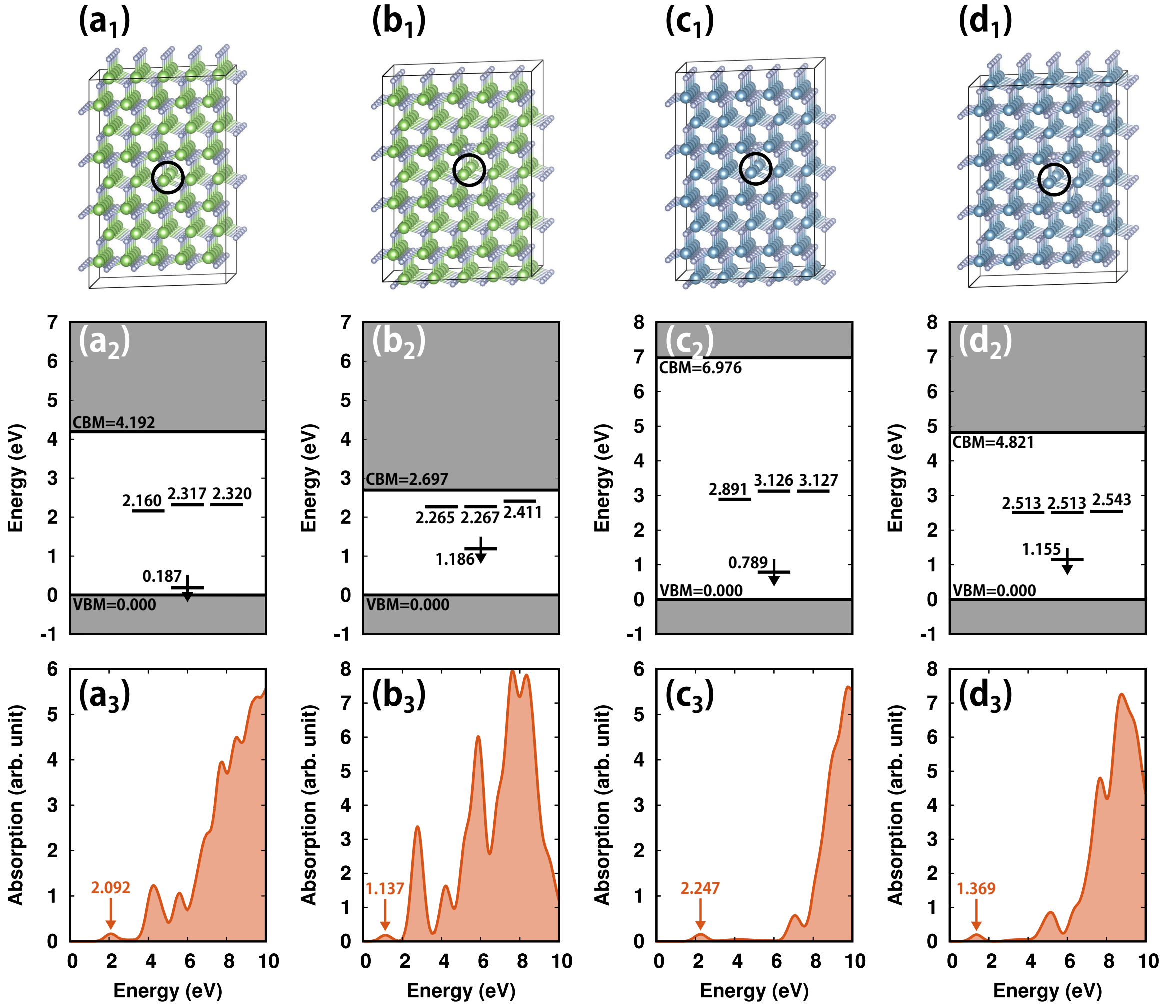

Figure S9: (a_1)-(d_1) are the atomic supercell structures for V_{Ga} in 95%strain-GaN, V_{Ga} in 105%strain-GaN, V_{Al} in 95%strain-AlN, and V_{Al} in 105%strain-AlN, the corresponding defect energy levels in the spin-down channel (the VBM is set to zero) and absorption spectrum calculated with HSE06 functional are shown in (a_2)-(d_2) and (a_3)-(d_3), respectively.

The defect energy level and absorption spectrum of V_{Ga} in AlGaN alloy calculated with HSE06 functional are shown in Figure S10. A 399-atom supercell is used for $Al_{0.25}Ga_{0.75}N$, a 359-atom supercell is used for $Al_{0.5}Ga_{0.5}N$, a 359-atom supercell is used for $Al_{0.8}Ga_{0.2}N$, all the calculation use a Γ point only K mesh sampling.

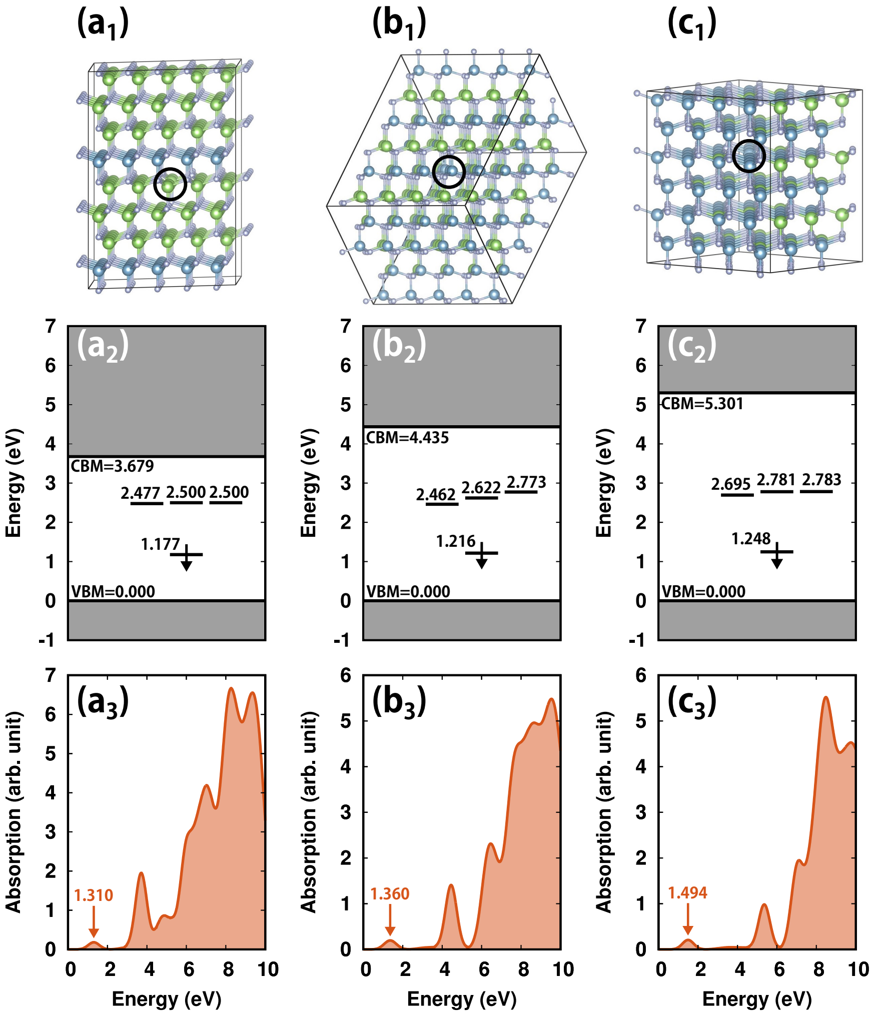

Figure S10: (a_1)-(c_1) are the atomic supercell structures for V_{Ga} in $Al_{0.25}Ga_{0.75}N$, $Al_{0.5}Ga_{0.5}N$, and $Al_{0.8}Ga_{0.2}N$, the corresponding defect energy levels in the spin-down channel (the VBM is set to zero) and absorption spectrum calculated with HSE06 functional are shown in (a_2)-(c_2) and (a_3)-(c_3), respectively.

3.3 Ground and excited state geometries of V_{cation}

The interatomic distance (in Å) between the four N atoms (index 1 to 4) around $V_{catoion}$ at ground (excited) states are depicted in Table S2, index 1 represents the N atom along the (0001) direction, index 2-4 represent the remaining N atoms.

Table S2: Distances between N atoms around the V_{cation} in GaN, AlN, AlGaN, and InGaN at ground (excited) states.

	1-2	1-3	1-4	2-3	2-4	3-4
V_{Ga} in 95%stain-GaN	3.61(3.68)	3.61(3.68)	3.61(3.68)	3.62(3.72)	3.62(3.74)	3.62(3.72)
V_{Ga} in GaN	3.61(3.68)	3.61(3.68)	3.61(3.68)	3.62(3.72)	3.62(3.74)	3.62(3.72)
V_{Ga} in 105%stain-GaN	3.83(3.91)	3.83(3.91)	3.83(3.91)	4.06(4.13)	4.06(4.14)	4.06(4.13)
V_{Al} in $Al_{0.25}Ga_{0.75}N$	3.71(3.80)	3.71(3.78)	3.71(3.78)	3.83(3.90)	3.83(3.89)	3.83(3.88)
V_{Ga} in $Al_{0.25}Ga_{0.75}N$	3.75(3.80)	3.75(3.81)	3.75(3.80)	3.70(3.80)	3.70(3.81)	3.70(3.80)
V_{Al} in $Al_{0.5}Ga_{0.5}N$	3.62(3.71)	3.62(3.72)	3.69(3.77)	3.70(3.81)	3.64(3.72)	3.63(3.72)
V_{Ga} in $Al_{0.5}Ga_{0.5}N$	3.73(3.82)	3.65(3.75)	3.65(3.75)	3.71(3.79)	3.71(3.79)	3.62(3.73)
V_{Al} in $Al_{0.8}Ga_{0.2}N$	3.54(3.65)	3.56(3.64)	3.56(3.66)	3.59(3.68)	3.57(3.64)	3.62(3.70)
V_{Ga} in $Al_{0.8}Ga_{0.2}N$	3.59(3.70)	3.59(3.70)	3.58(3.67)	3.63(3.70)	3.63(3.72)	3.62(3.71)
V_{Al} in 95%stain-AlN	3.25(3.23)	3.25(3.23)	3.25(3.23)	3.05(3.05)	3.05(3.04)	3.05(3.05)
V_{Al} in AlN	3.46(3.54)	3.46(3.58)	3.46(3.54)	3.51(3.59)	3.51(3.59)	3.51(3.59)
V_{Al} in 105%stain-AlN	3.65(3.72)	3.65(3.69)	3.65(3.72)	3.92(3.97)	3.92(3.97)	3.92(3.97)
V_{In} in $In_{0.125}Ga_{0.875}N$	3.97(3.99)	3.91(3.96)	3.91(3.96)	3.88(3.97)	3.88(3.97)	3.40(3.50)
V_{Ga} in $In_{0.125}Ga_{0.875}N$	3.75(3.85)	4.02(4.08)	3.75(3.85)	3.92(4.01)	3.90(4.03)	3.92(4.01)

3.4 Moment matrix for V_{cation}

The calculated moment matrices $|\langle\psi_i|p|\psi_j\rangle|^2$ (in atomic unit) of V_{cation} in III-nitrides (corresponding to Table 1) among VBM, CBM, and all the defect levels in the spin-down channel are shown below.

V_{Ga}@95% − GaN	VBM	A_1	$T_2(1)$	$T_2(2)$	$T_2(3)$	CBM
VBM	3.97×10^{-14}	1.02×10^{-03}	3.24×10^{-05}	5.21×10^{-05}	5.33×10^{-05}	3.91×10^{-01}
A_1	1.02×10^{-03}	1.72×10^{-14}	1.90×10^{-02}	2.28×10^{-02}	2.27×10^{-02}	3.07×10^{-04}
$T_2(1)$	3.24×10^{-05}	1.90×10^{-02}	8.96×10^{-15}	2.68×10^{-05}	2.72×10^{-05}	6.77×10^{-03}
$T_2(2)$	5.21×10^{-05}	2.28×10^{-02}	2.68×10^{-05}	1.37×10^{-14}	1.47×10^{-08}	7.15×10^{-03}
$T_2(3)$	5.33×10^{-05}	2.27×10^{-02}	2.72×10^{-05}	1.47×10^{-08}	5.91×10^{-15}	7.14×10^{-03}
CBM	3.91×10^{-01}	3.07×10^{-04}	6.77×10^{-03}	7.15×10^{-03}	7.14×10^{-03}	2.08×10^{-14}

(S14)

V_{Ga}@GaN	VBM	A_1	$T_2(1)$	$T_2(2)$	$T_2(3)$	CBM
VBM	7.15×10^{-14}	8.17×10^{-04}	1.60×10^{-05}	1.70×10^{-05}	7.35×10^{-05}	4.06×10^{-01}
A_1	8.17×10^{-04}	2.65×10^{-13}	1.05×10^{-02}	1.05×10^{-02}	1.60×10^{-02}	8.54×10^{-06}
$T_2(1)$	1.60×10^{-05}	1.05×10^{-02}	6.73×10^{-13}	1.64×10^{-10}	1.11×10^{-04}	4.72×10^{-03}
$T_2(2)$	1.70×10^{-05}	1.05×10^{-02}	1.64×10^{-10}	1.36×10^{-13}	1.11×10^{-04}	4.72×10^{-03}
$T_2(3)$	7.35×10^{-05}	1.60×10^{-02}	1.11×10^{-04}	1.11×10^{-04}	5.83×10^{-13}	5.30×10^{-03}
CBM	4.06×10^{-01}	8.54×10^{-06}	4.72×10^{-03}	4.72×10^{-03}	5.30×10^{-03}	1.06×10^{-14}

(S15)

V_{Ga}@105% − GaN	VBM	A_1	$T_2(1)$	$T_2(2)$	$T_2(3)$	CBM
VBM	6.88×10^{-16}	7.72×10^{-04}	2.41×10^{-05}	2.41×10^{-05}	1.40×10^{-03}	3.78×10^{-01}
A_1	7.72×10^{-04}	1.14×10^{-12}	6.06×10^{-03}	6.05×10^{-03}	1.27×10^{-02}	1.09×10^{-04}
$T_2(1)$	2.41×10^{-05}	6.06×10^{-03}	3.62×10^{-13}	2.93×10^{-08}	2.62×10^{-04}	3.48×10^{-03}
$T_2(2)$	2.41×10^{-05}	6.05×10^{-03}	2.93×10^{-08}	3.24×10^{-13}	2.62×10^{-04}	3.48×10^{-03}
$T_2(3)$	1.40×10^{-03}	1.27×10^{-02}	2.62×10^{-04}	2.62×10^{-04}	2.29×10^{-13}	4.34×10^{-03}
CBM	3.78×10^{-01}	1.09×10^{-04}	3.48×10^{-03}	3.48×10^{-03}	4.34×10^{-03}	1.74×10^{-14}

(S16)

$V_{Al}@Al_{0.25}Ga_{0.75}N$	VBM	A_1	$T_2(1)$	$T_2(2)$	$T_2(3)$	CBM
VBM	9.28×10^{-15}	1.08×10^{-04}	2.23×10^{-05}	3.46×10^{-05}	2.67×10^{-04}	3.52×10^{-01}
A_1	1.08×10^{-04}	1.92×10^{-13}	9.22×10^{-03}	9.21×10^{-03}	1.47×10^{-02}	1.92×10^{-05}
$T_2(1)$	2.23×10^{-05}	9.22×10^{-03}	8.60×10^{-13}	1.01×10^{-09}	3.38×10^{-04}	2.21×10^{-03}
$T_2(2)$	3.46×10^{-05}	9.21×10^{-03}	1.01×10^{-09}	4.13×10^{-13}	3.37×10^{-04}	2.21×10^{-03}
$T_2(3)$	2.67×10^{-04}	1.47×10^{-02}	3.38×10^{-04}	3.37×10^{-04}	6.44×10^{-13}	4.59×10^{-03}
CBM	3.52×10^{-01}	1.92×10^{-05}	2.21×10^{-03}	2.21×10^{-03}	4.59×10^{-03}	2.82×10^{-14}

(S17)

$V_{Ga}@Al_{0.25}Ga_{0.75}N$	VBM	A_1	$T_2(1)$	$T_2(2)$	$T_2(3)$	CBM
VBM	5.26×10^{-14}	4.43×10^{-04}	3.77×10^{-05}	2.58×10^{-06}	2.74×10^{-06}	3.57×10^{-01}
A_1	4.43×10^{-04}	3.01×10^{-13}	1.31×10^{-02}	8.54×10^{-03}	8.54×10^{-03}	4.86×10^{-05}
$T_2(1)$	3.77×10^{-05}	1.31×10^{-02}	3.71×10^{-14}	7.01×10^{-05}	7.03×10^{-05}	4.33×10^{-03}
$T_2(2)$	2.58×10^{-06}	8.54×10^{-03}	7.01×10^{-05}	4.06×10^{-13}	5.38×10^{-09}	3.88×10^{-03}
$T_2(3)$	2.74×10^{-06}	8.54×10^{-03}	7.03×10^{-05}	5.38×10^{-09}	1.54×10^{-12}	3.88×10^{-03}
CBM	3.57×10^{-01}	4.86×10^{-05}	4.33×10^{-03}	3.88×10^{-03}	3.88×10^{-03}	2.28×10^{-14}

(S18)

$$\left(\begin{array}{c|cccccc}
V_{Al}@Al_{0.5}Ga_{0.5}N & VBM & A_1 & T_2(1) & T_2(2) & T_2(3) & CBM \\ \hline
VBM & 4.15\times10^{-14} & 4.83\times10^{-04} & 4.39\times10^{-05} & 9.22\times10^{-06} & 1.37\times10^{-04} & 3.94\times10^{-01} \\
A_1 & 4.83\times10^{-04} & 1.04\times10^{-13} & 1.61\times10^{-02} & 1.21\times10^{-02} & 1.39\times10^{-02} & 1.85\times10^{-04} \\
T_2(1) & 4.39\times10^{-05} & 1.61\times10^{-02} & 4.57\times10^{-14} & 2.22\times10^{-06} & 2.23\times10^{-04} & 4.32\times10^{-03} \\
T_2(2) & 9.22\times10^{-06} & 1.21\times10^{-02} & 2.22\times10^{-06} & 2.11\times10^{-13} & 2.14\times10^{-04} & 4.32\times10^{-03} \\
T_2(3) & 1.37\times10^{-04} & 1.39\times10^{-02} & 2.23\times10^{-04} & 2.14\times10^{-04} & 1.90\times10^{-13} & 4.73\times10^{-03} \\
CBM & 3.94\times10^{-01} & 1.85\times10^{-04} & 4.32\times10^{-03} & 4.32\times10^{-03} & 4.73\times10^{-03} & 1.35\times10^{-15}
\end{array}\right) \tag{S19}$$

$$\left(\begin{array}{c|cccccc}
V_{Ga}@Al_{0.5}Ga_{0.5}N & VBM & A_1 & T_2(1) & T_2(2) & T_2(3) & CBM \\ \hline
VBM & 1.13\times10^{-15} & 2.74\times10^{-04} & 1.74\times10^{-05} & 3.07\times10^{-06} & 5.48\times10^{-05} & 3.94\times10^{-01} \\
A_1 & 2.74\times10^{-04} & 2.63\times10^{-14} & 1.31\times10^{-02} & 1.47\times10^{-02} & 1.15\times10^{-02} & 4.19\times10^{-05} \\
T_2(1) & 1.74\times10^{-05} & 1.31\times10^{-02} & 5.97\times10^{-13} & 1.91\times10^{-04} & 1.03\times10^{-05} & 3.19\times10^{-03} \\
T_2(2) & 3.07\times10^{-06} & 1.47\times10^{-02} & 1.91\times10^{-04} & 6.67\times10^{-14} & 9.47\times10^{-07} & 3.50\times10^{-03} \\
T_2(3) & 5.48\times10^{-05} & 1.15\times10^{-02} & 1.03\times10^{-05} & 9.47\times10^{-07} & 1.11\times10^{-12} & 4.49\times10^{-03} \\
CBM & 3.94\times10^{-01} & 4.19\times10^{-05} & 3.19\times10^{-03} & 3.50\times10^{-03} & 4.49\times10^{-03} & 4.17\times10^{-15}
\end{array}\right) \tag{S20}$$

$$\left(\begin{array}{c|cccccc}
V_{Al}@Al_{0.8}Ga_{0.2}N & VBM & A_1 & T_2(1) & T_2(2) & T_2(3) & CBM \\ \hline
VBM & 6.44\times10^{-15} & 3.92\times10^{-04} & 2.15\times10^{-05} & 6.63\times10^{-06} & 1.46\times10^{-05} & 4.28\times10^{-01} \\
A_1 & 3.92\times10^{-04} & 1.95\times10^{-13} & 1.60\times10^{-02} & 1.63\times10^{-02} & 1.48\times10^{-02} & 1.36\times10^{-04} \\
T_2(1) & 2.15\times10^{-05} & 1.60\times10^{-02} & 3.54\times10^{-13} & 2.29\times10^{-05} & 1.02\times10^{-05} & 4.19\times10^{-03} \\
T_2(2) & 6.63\times10^{-06} & 1.63\times10^{-02} & 2.29\times10^{-05} & 2.69\times10^{-14} & 6.47\times10^{-05} & 4.53\times10^{-03} \\
T_2(3) & 1.46\times10^{-05} & 1.48\times10^{-02} & 1.02\times10^{-05} & 6.47\times10^{-05} & 7.98\times10^{-13} & 3.98\times10^{-03} \\
CBM & 4.28\times10^{-01} & 1.36\times10^{-04} & 4.19\times10^{-03} & 4.53\times10^{-03} & 3.98\times10^{-03} & 4.82\times10^{-15}
\end{array}\right) \tag{S21}$$

$$\left(\begin{array}{c|cccccc}
V_{Ga}@Al_{0.8}Ga_{0.2}N & VBM & A_1 & T_2(1) & T_2(2) & T_2(3) & CBM \\ \hline
VBM & 4.30\times10^{-16} & 1.60\times10^{-04} & 1.18\times10^{-05} & 8.87\times10^{-06} & 6.27\times10^{-06} & 4.29\times10^{-01} \\
A_1 & 1.60\times10^{-04} & 2.58\times10^{-13} & 1.71\times10^{-02} & 1.39\times10^{-02} & 1.37\times10^{-02} & 7.92\times10^{-05} \\
T_2(1) & 1.18\times10^{-05} & 1.71\times10^{-02} & 1.21\times10^{-13} & 4.93\times10^{-05} & 4.31\times10^{-05} & 3.15\times10^{-03} \\
T_2(2) & 8.87\times10^{-06} & 1.39\times10^{-02} & 4.93\times10^{-05} & 1.27\times10^{-13} & 2.65\times10^{-07} & 2.99\times10^{-03} \\
T_2(3) & 6.27\times10^{-06} & 1.37\times10^{-02} & 4.31\times10^{-05} & 2.65\times10^{-07} & 2.02\times10^{-13} & 3.10\times10^{-03} \\
CBM & 4.29\times10^{-01} & 7.92\times10^{-05} & 3.15\times10^{-03} & 2.99\times10^{-03} & 3.10\times10^{-03} & 2.87\times10^{-16}
\end{array}\right) \tag{S22}$$

$$\left(\begin{array}{c|cccccc}
V_{Al}@95\%-AlN & VBM & A_1 & T_2(1) & T_2(2) & T_2(3) & CBM \\ \hline
VBM & 2.42\times10^{-15} & 2.64\times10^{-04} & 1.53\times10^{-07} & 6.23\times10^{-05} & 6.26\times10^{-05} & 4.17\times10^{-01} \\
A_1 & 2.64\times10^{-04} & 1.09\times10^{-14} & 2.59\times10^{-02} & 2.64\times10^{-02} & 2.64\times10^{-02} & 8.56\times10^{-07} \\
T_2(1) & 1.53\times10^{-07} & 2.59\times10^{-02} & 6.37\times10^{-16} & 4.15\times10^{-06} & 4.16\times10^{-06} & 4.16\times10^{-03} \\
T_2(2) & 6.23\times10^{-05} & 2.64\times10^{-02} & 4.15\times10^{-06} & 2.46\times10^{-14} & 1.33\times10^{-09} & 4.50\times10^{-03} \\
T_2(3) & 6.26\times10^{-05} & 2.64\times10^{-02} & 4.16\times10^{-06} & 1.33\times10^{-09} & 4.01\times10^{-15} & 4.50\times10^{-03} \\
CBM & 4.17\times10^{-01} & 8.56\times10^{-07} & 4.16\times10^{-03} & 4.50\times10^{-03} & 4.50\times10^{-03} & 3.01\times10^{-16}
\end{array}\right) \tag{S23}$$

V_{Al}@AlN	VBM	A_1	$T_2(1)$	$T_2(2)$	$T_2(3)$	CBM
VBM	1.12×10^{-15}	3.30×10^{-04}	1.04×10^{-05}	1.31×10^{-05}	1.31×10^{-05}	4.42×10^{-01}
A_1	3.30×10^{-04}	8.28×10^{-15}	2.24×10^{-02}	1.36×10^{-02}	1.35×10^{-02}	3.87×10^{-05}
$T_2(1)$	1.04×10^{-05}	2.24×10^{-02}	1.96×10^{-14}	1.15×10^{-05}	1.15×10^{-05}	4.32×10^{-03}
$T_2(2)$	1.31×10^{-05}	1.36×10^{-02}	1.15×10^{-05}	3.73×10^{-15}	7.40×10^{-10}	3.33×10^{-03}
$T_2(3)$	1.31×10^{-05}	1.35×10^{-02}	1.15×10^{-05}	7.40×10^{-10}	2.21×10^{-15}	3.33×10^{-03}
CBM	4.42×10^{-01}	3.87×10^{-05}	4.32×10^{-03}	3.33×10^{-03}	3.33×10^{-03}	1.19×10^{-15}

(S24)

V_{Al}@$105\% - AlN$	VBM	A_1	$T_2(1)$	$T_2(2)$	$T_2(3)$	CBM
VBM	2.73×10^{-16}	2.78×10^{-04}	2.54×10^{-05}	2.54×10^{-05}	2.90×10^{-05}	3.74×10^{-01}
A_1	2.78×10^{-04}	6.04×10^{-14}	8.54×10^{-03}	8.53×10^{-03}	1.85×10^{-02}	6.71×10^{-05}
$T_2(1)$	2.54×10^{-05}	8.54×10^{-03}	1.58×10^{-14}	1.40×10^{-09}	5.08×10^{-05}	2.59×10^{-03}
$T_2(2)$	2.54×10^{-05}	8.53×10^{-03}	1.40×10^{-09}	8.21×10^{-15}	5.10×10^{-05}	2.59×10^{-03}
$T_2(3)$	2.90×10^{-05}	1.85×10^{-02}	5.08×10^{-05}	5.10×10^{-05}	6.16×10^{-13}	4.15×10^{-03}
CBM	3.74×10^{-01}	6.71×10^{-05}	2.59×10^{-03}	2.59×10^{-03}	4.15×10^{-03}	6.47×10^{-16}

(S25)

V_{In}@$In_{0.125}Ga_{0.875}N$	VBM	A_1	$T_2(1)$	$T_2(2)$	$T_2(3)$	CBM
VBM	8.26×10^{-15}	1.35×10^{-03}	1.65×10^{-04}	9.72×10^{-04}	2.16×10^{-05}	2.75×10^{-01}
A_1	1.35×10^{-03}	1.29×10^{-12}	7.38×10^{-03}	8.72×10^{-03}	8.74×10^{-03}	3.05×10^{-04}
$T_2(1)$	1.65×10^{-04}	7.38×10^{-03}	1.71×10^{-12}	4.06×10^{-04}	5.06×10^{-05}	5.48×10^{-03}
$T_2(2)$	9.72×10^{-04}	8.72×10^{-03}	4.06×10^{-04}	5.51×10^{-12}	3.85×10^{-05}	6.15×10^{-03}
$T_2(3)$	2.16×10^{-05}	8.74×10^{-03}	5.06×10^{-05}	3.85×10^{-05}	3.18×10^{-13}	6.37×10^{-03}
CBM	2.75×10^{-01}	3.05×10^{-04}	5.48×10^{-03}	6.15×10^{-03}	6.37×10^{-03}	3.13×10^{-13}

(S26)

V_{Ga}@$In_{0.125}Ga_{0.875}N$	VBM	A_1	$T_2(1)$	$T_2(2)$	$T_2(3)$	CBM
VBM	1.56×10^{-14}	9.64×10^{-04}	6.95×10^{-05}	1.08×10^{-03}	8.48×10^{-05}	2.94×10^{-01}
A_1	9.64×10^{-04}	1.67×10^{-12}	4.48×10^{-03}	8.96×10^{-03}	8.47×10^{-03}	2.94×10^{-04}
$T_2(1)$	6.95×10^{-05}	4.48×10^{-03}	6.08×10^{-14}	2.01×10^{-05}	2.22×10^{-03}	5.88×10^{-03}
$T_2(2)$	1.08×10^{-03}	8.96×10^{-03}	2.01×10^{-05}	1.94×10^{-12}	1.28×10^{-04}	6.12×10^{-03}
$T_2(3)$	8.48×10^{-05}	8.47×10^{-03}	2.22×10^{-03}	1.28×10^{-04}	1.76×10^{-12}	5.61×10^{-03}
CBM	2.94×10^{-01}	2.94×10^{-04}	5.88×10^{-03}	6.12×10^{-03}	5.61×10^{-03}	1.28×10^{-13}

(S27)

References

[1] A. van de Walle, M. Asta, G. Ceder, *CALPHAD: Comput. Coupling Phase Diagrams Thermochem.* **2002**, *26* 539.

[2] A. Walle, G. Ceder, *J. Phase Equilib.* **2002**, *23* 348.

[3] J. P. Perdew, K. Burke, M. Ernzerhof, *Phys. Rev. Lett.* **1996**, *77* 3865.

[4] G. Kresse, J. Furthmüller, *Phys. Rev. B* **1996**, *54* 11169.

[5] G. Kresse, J. Furthmüller, *Comput. Mater. Sci.* **1996**, *6* 15.

[6] J. Heyd, G. E. Scuseria, M. Ernzerhof, *J. Chem. Phys.* **2003**, *118* 8207.

[7] W. Jia, Z. Cao, L. Wang, J. Fu, X. Chi, W. Gao, L.-W. Wang, *Comput. Phys. Commun.* **2013**, *184* 9.

[8] W. Jia, J. Fu, Z. Cao, L. Wang, X. Chi, W. Gao, L.-W. Wang, *J. Comput. Phys.* **2013**, *251* 102.

[9] A. Alkauskas, P. Broqvist, A. Pasquarello, *Phys. Rev. Lett.* **2008**, *101* 046405.

[10] D. R. Hamann, *Phys. Rev. B* **2013**, *88* 085117.

[11] B. Monemar, *Phys. Rev. B* **1974**, *10* 676.

[12] I. Vurgaftman, J. R. Meyer, L. R. Ram-Mohan, *J. Appl. Phys.* **2001**, *89* 5815.

[13] M. Suzuki, T. Uenoyama, A. Yanase, *Phys. Rev. B* **1995**, *52* 8132.

[14] S.-H. Wei, A. Zunger, *Appl. Phys. Lett.* **1996**, *69* 2719.

[15] A. F. van Driel, G. Allan, C. Delerue, P. Lodahl, W. L. Vos, D. Vanmaekelbergh, *Phys. Rev. Lett.* **2005**, *95* 236804.

[16] S.-H. Wei, *Comput. Mater. Sci.* **2004**, *30* 337.
